# BARTweb: a web server for transcriptional regulator association analysis

**DOI:** 10.1093/nargab/lqab022

**Published:** 2021-04-09

**Authors:** Wenjing Ma, Zhenjia Wang, Yifan Zhang, Neal E Magee, Yayi Feng, Ruoyao Shi, Yang Chen, Chongzhi Zang

**Affiliations:** Center for Public Health Genomics, University of Virginia, Charlottesville, VA 22908, USA; Center for Public Health Genomics, University of Virginia, Charlottesville, VA 22908, USA; Center for Public Health Genomics, University of Virginia, Charlottesville, VA 22908, USA; Research Computing, University of Virginia, Charlottesville, VA 22903, USA; Center for Public Health Genomics, University of Virginia, Charlottesville, VA 22908, USA; Center for Public Health Genomics, University of Virginia, Charlottesville, VA 22908, USA; Department of Statistics, University of Michigan, Ann Arbor, MI 48109, USA; Center for Public Health Genomics, University of Virginia, Charlottesville, VA 22908, USA; Department of Public Health Sciences, University of Virginia, Charlottesville, VA 22908, USA

## Abstract

Identifying active transcriptional regulators (TRs) associating with *cis*-regulatory elements in the genome to regulate gene expression is a key task in gene regulation research. TR binding profiles from numerous public ChIP-seq data can be utilized for association analysis with query data for TR identification, as an alternative to DNA sequence motif analysis. However, integration of the massive ChIP-seq datasets has been a major challenge in such approaches. Here we present BARTweb, an interactive web server for identifying TRs whose genomic binding patterns associate with input genomic features, by leveraging over 13 000 public ChIP-seq datasets for human and mouse. Using an updated binding analysis for regulation of transcription (BART) algorithm, BARTweb can identify functional TRs that regulate a gene set, have a binding profile correlated with a ChIP-seq profile or are enriched in a genomic region set, without *a priori* information of the cell type. BARTweb can be a useful web server for performing functional analysis of gene regulation. BARTweb is freely available at http://bartweb.org and the source code is available at https://github.com/zanglab/bart2.

## INTRODUCTION

Transcriptional regulators (TRs), including DNA sequence-specific transcription factors (TFs) and chromatin regulators, play an instrumental role in controlling gene expression by interacting with DNA and chromatin in the eukaryotic genome (1). An important task in gene regulation studies is to identify active TRs that function to regulate genes with differential expression or are enriched for binding in certain regions in the genome. Chromatin immunoprecipitation followed by high-throughput sequencing (ChIP-seq) has become one of the most commonly used techniques for genome-wide profiling of TR binding sites and chromatin marks (2,3). The increasing amount of publicly available ChIP-seq datasets generated by individual laboratories worldwide as well as large collaborating consortia, such as Encyclopedia of DNA Elements (ENCODE) (4) and Roadmap Epigenomics (5) is a valuable resource for interrogating genomic profiles for hundreds of TRs in many human and mouse cell types (6). As an alternative to DNA-binding sequence motif search, ChIP-seq data collected from the public domain can be utilized to perform TR analysis.

To leverage public ChIP-seq data for TR identification, we previously developed binding analysis for regulation of transcription (BART), an algorithm to identify TRs from a large collection of ChIP-seq data that have a genomic binding pattern highly correlated with an input genomic profile, using a novel statistical approach integrating multiple levels of statistical tests (7). To infer TRs regulating a query gene set, BART first applies model-based analysis of regulation of gene expression (MARGE) (8) to derive a genomic *cis*-regulatory profile from the input gene set leveraging compendium ChIP-seq data for active enhancer histone mark H3K27ac, and then generates a ranked list of factors that have a highly correlated binding profile with the *cis*-regulatory profile. While proven to work for identifying functional TRs in many case studies (7,9–14), BART requires users to download large ChIP-seq data libraries that can be storage and memory consuming, and sometimes runs slow primarily due to the stepwise regression computation in MARGE.

Besides conventional sequence motif-based methods, such as HOMER (15) and Pscan (16), there are several other bioinformatics tools that use existing ChIP-seq data for TR identification or enrichment analysis, including ChIP-Atlas (17), TFEA.ChIP (18) and ChEA3 (19). ChIP-Atlas applies the Fisher's exact test for TR enrichment near a gene locus using collected public ChIP-seq data from multiple resources. TFEA.ChIP applies the Fisher's exact test or the gene set enrichment analysis method (20) for TR enrichment analysis using ChIP-seq data collected from the ReMap database (21). ChEA3 integrates multiple sources of TR–target association information including ChIP-seq, co-expression from RNA-seq and collected crowd-based gene lists (22) to generate a ranked list of TRs associated with query gene sets.

To overcome several data-intensive computing burdens and to improve the performance of the original BART package, we present BARTweb, a web server application for users to perform TR analysis from multiple types of query data. BARTweb is accessible through an interactive web interface, from which users can submit jobs and obtain results including a table of TRs with statistical assessments and several analysis plots for each factor. BARTweb implements an updated BART algorithm for faster and more robust performances. We demonstrate that BARTweb outperforms several existing tools in identifying true TRs from collected experimental data, and can be a useful tool for gene regulation research.

## MATERIALS AND METHODS

### BARTweb server infrastructure design

**Figure 1.**
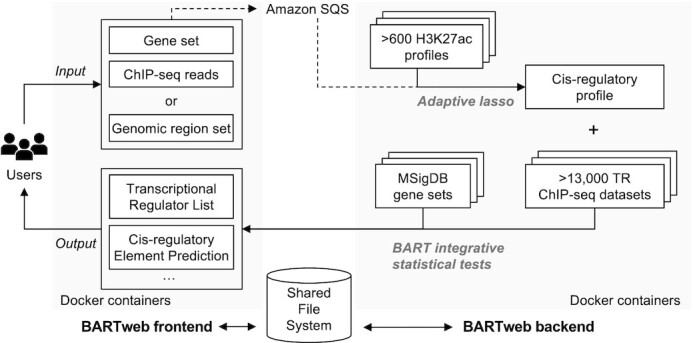
BARTweb architecture overview. BARTweb front-end receives user input and displays processed output. BARTweb back-end performs computation of BART TR identification analysis. Both services are containerized and share a common file system.

To provide a user-friendly and stable service through web interface, we designed a two-part structure for the BARTweb server: a front-end web interface to receive users’ job submission requests and to display job execution information and results; and a back-end computing service to perform all computation (Figure 1). We containerized both parts into Docker and deployed them on a 17-server Distributed Cloud Operating System cluster for continuous and stable services.

The front-end web interface was developed and implemented in Flask. To support simultaneous users, we deployed it under Apache 2.4 inside a Docker container. The back-end service uses our updated BART algorithm implemented in Python3. To ensure continuous deployment of the website, we serialized both parts into a GitHub repository and use Travis Continuous Integration to automatically push the code changes into the online environment running in production.

To connect the front-end and the back-end and to scale to many users, we employed a robust queue using Amazon's Simple Queue Service to temporarily store job keys. Every time a user submits a new job request through the web interface, the BARTweb front-end pushes a unique message to that request into the queue. Meanwhile, the BARTweb back-end routinely checks that queue for incoming requests, executes as soon as a new job comes in, and removes the request from the queue.

### Updated BART algorithm

In BARTweb, we implemented an updated BART algorithm, in which the inference of the genomic *cis*-regulatory profile from the input gene set by integrating compendium H3K27ac ChIP-seq data was replaced from the original MARGE algorithm (8) with the adaptive lasso (23). MARGE adopts a forward stepwise regression for feature selection to identify significant predictors, i.e. informative H3K27ac profiles that carry regulatory potential (RP) information to better separate the input gene set from other genes in the genome. However, stepwise regression has fundamental limitations including selecting extremely variable features and frequently trapped into a local optimal solution. In addition, *k*-fold cross-validation makes the entire job execution very slow. The adaptive lasso can solve these issues for this feature selection process.

Similar to MARGE, we consider the selection of informative H3K27ac samples as a logistic regression model. Suppose ${\boldsymbol{y\ }} = {( {{y_1}, \ldots {y_n}} )^T}$ be the response vector indicating whether a gene belongs to a given gene set or not, and ${\boldsymbol{P\ }} = {\mathrm{\ }}[ {{{\boldsymbol{p}}_1},{\mathrm{\ }} \ldots {{\boldsymbol{p}}_m}} ]$ be the predictor matrix, i.e. the normalized RP matrix derived from H3K27ac profiles (8). We assume that:


\begin{equation*}E\left[ {y|{\boldsymbol{P}}} \right] = {\boldsymbol{\ }}1/\left( {1 + {e^{ - \ \left( {\beta _1{{\boldsymbol{p}}_1} + \ldots + \beta _m{{\boldsymbol{p}}_m}} \right)}}} \right)\end{equation*}

where *β* is the estimated value of each coefficient. We further assume $|\{ j:\ \beta _j \ne 0\} |{\boldsymbol{\ }} = {m_0}\ \ < m$, and the model to estimate the input gene set depends only on a sparse representation of the predictors, i.e., a small subset of samples from the H3K27ac ChIP-seq data compendium. We use adaptive lasso to identify an accurate sparse representation of the predictors. The generalized logistic adaptive lasso is defined as:


\begin{eqnarray*} {\boldsymbol{\hat{\beta }}}({{\rm logistic}-{\rm adalasso}}) &=& \mathop {{\mathrm{arg\ min}}}\limits_{\boldsymbol{\beta }} \ \mathop \sum \limits_{i = 1}^n \left( { - {y_i}\left( {{\boldsymbol{p}}_i^T{\boldsymbol{\beta }}} \right) + {\mathrm{log}}\left( {1 + {e^{{\boldsymbol{p}}_i^T{\boldsymbol{\beta }}}}} \right)} \right) \\ &&+ \lambda \mathop \sum \limits_{j = 1}^m {\hat{w}_j}\left| {{\beta _j}} \right|\ \end{eqnarray*}

where *w* is the adaptive weight used for penalizing each coefficient. The adaptive lasso carries the oracle properties, namely, it can simultaneously achieve consistent variable selection and optimal prediction rate. Compared to lasso, which equally penalizes the coefficients in the ${\mathfrak{l}_1}$ penalty, adaptive lasso uses data-dependent adaptive weights to penalize different coefficients in the ${\mathfrak{l}_1}$ penalty. The weight vector can be selected based on the importance of different indicators so that large and important coefficients are not penalized much and irrelevant variables are penalized more. By performing a different regularization for each coefficient, the adaptive lasso avoids over-penalization of relevant coefficients, reduces the estimation biases and leads to a consistent model selection (24). Besides, by applying the LARS algorithm (25) that is implemented in our model, the adaptive lasso is in the same order of computation of a single ordinary least squares fit (23).

If the weights are cleverly chosen, the adaptive lasso performs equally well as if the true underlying model were given in advance (23). Here, we iteratively construct the data-dependent adaptive weights. The weights are initiated as an all-one-vector, and then are iteratively determined by the coefficient from the logistic lasso in the previous step. The algorithm is described in Supplementary Algorithm 1.

After relevant H3K27ac samples are selected, we directly apply the feature coefficients on the H3K27ac signals and produce a score for each candidate cis-regulatory element (CRE). Here we use the union DNase hypersensitive sites (UDHS) as a collection of all candidate CREs. The higher the score is, the more likely this CRE is a functional element regulating the input gene set. All candidate CREs with prediction scores compose the genomic *cis*-regulatory profile, which undergoes the remaining steps in the BART algorithm.

### Updated ChIP-seq data library

The amount of available ChIP-seq data keeps growing in the public domain. We updated the ChIP-seq data library to cover more TRs in more cell types for both human and mouse. We downloaded the TR ChIP-seq peak files from the updated Cistrome Data Browser (26). Under the same quality control standards used in BART v1.1 (7), we kept only the datasets that have at least 2000 peaks. The updated data library contains 7968 ChIP-seq datasets for 918 human TRs and 5851 ChIP-seq datasets for 565 mouse TRs, a significant increase from BART 1.1 (Supplementary Table S1 and Figure S1). We plan to keep updating the data library regularly.

## RESULTS

### Submit jobs on BARTweb

The web server interface is shown in Supplementary Figure S2. When submitting a job through the web interface, users need to specify the species (hg38 for human or mm10 for mouse) and the input data type (a gene set, a ChIP-seq dataset or a scored region set) besides providing the input data. The input data can be either uploaded as a file in an accepted format, or pasted in the input field. Users can opt to assign a job name and/or provide an email address. Once a job is submitted, BARTweb will generate a unique key, and display a status indicator and a processing log. It usually takes a few minutes to run a job. Users can leave BARTweb running in the background, and use the unique key or the provided email to check the job status and to retrieve the results with a uniform resource locator (URL). The results are kept on the server with the unique keys or URLs for a minimum of 180 days.

### BARTweb input

BARTweb accepts three data types as input:

- *a gene set* in official gene symbols (HGNC for human or MGI for mouse) in text format. BARTweb will identify TRs that regulate this gene set. BARTweb will integrate the gene set with H3K27ac ChIP-seq data compendium to derive a genomic *cis*-regulatory profile. TR association analysis is performed on this genomic *cis*-regulatory profile. At least 100 genes are recommended in the input.
- *a ChIP-seq mapped read dataset* in BAM or BED format. BARTweb will identify TRs whose binding profile correlates with this ChIP-seq profile, e.g. co-factors of a TF or chromatin regulators associated with a TF or a histone mark. BARTweb will pile up the ChIP-seq reads located at the UDHS, and use the read count at each UDHS site to generate the genomic regulatory profile and to perform TR association analysis. At least 1 million reads are recommended in the input.
- *a scored genomic region set* in BED format. BARTweb will identify TRs with binding sites enriched in these genomic regions. BARTweb will map the region set to UDHS, and assign the region score to each UDHS overlapped with the region to generate the genomic regulatory profile and to perform TR association analysis. At least 1000 regions are recommended in the input.

### BARTweb output

**Figure 2.**
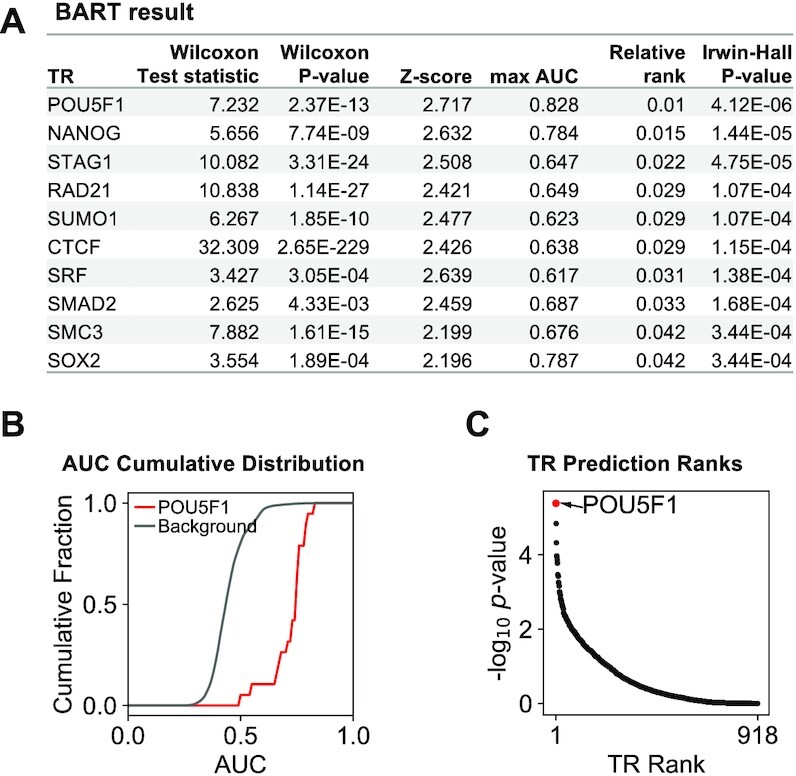
Example of BARTweb results. (**A**) Ranked list of identified TRs with quantification scores. (**B**) Cumulative distribution of association scores (AUC) of all ChIP-seq datasets for POU5F1 (red) compared with that of all other ChIP-seq datasets as background (gray). (**C**) Scatter plot of all TRs’ Irwin-Hall *P*-value score (−log_10_*P*-value) against its rank. Selected TR (POU5F1) was labeled in red.

BARTweb displays the result panel, including a ranked list of all TRs with quantification scores (Figure 2A) and a list of all intermediate and final output data files available for download. For each TR, clicking on the TR name can open a pop-up window displaying its corresponding analysis plots (Figure 2B and C).

In the output TR table (Figure 2A), all available TRs (918 for human or 565 for mouse) are displayed accompanied by six quantification scores in Columns 2–7. The table can be re-ordered by any score in a descending or ascending way by clicking the column header.

- *Wilcoxon test statistic* and *P-value* (Columns 2 and 3): these two values indicate the level of association of each TR under the background of all other TRs. For each TR, we use Wilcoxon rank-sum test to compare the association scores from all ChIP-seq datasets for that TR with the association scores from all ChIP-seq datasets for other TRs.
- *Z-score* (Column 4): this value is to assess the specificity of each TR compared with a background model. We build background models using the Wilcoxon test statistics obtained from all annotated gene sets from the Molecular Signatures Database (27) for gene set input or all H3K27ac ChIP-seq datasets from the data compendium for ChIP-seq read or region set input, respectively.
- *Max AUC* (Column 5): the maximum association score among multiple ChIP-seq datasets of that TR.
- *R* *elative rank* (Column 6): the average rank of Wilcoxon test statistic, *Z*-score and Max AUC for each TR, divided by the total number of TRs.
- *Irwin-Hall P-value* (Column 7): this *P*-value indicates the integrative rank significance, using the Irwin-Hall distribution as the null distribution for unrelated ranks. The output TRs are ranked by this *P*-value by default.

Example results shown in Figure 2 were generated using a gene set that were down-regulated upon OCT4 (POU5F1) knocked down in a human embryonic stem cell line. This input gene set should include target genes of POU5F1. As expected, POU5F1 was identified as the top ranked regulator, whereas several other stem cell signature TRs, such as NANOG and SOX2 were also identified.

Each TR in the table has a link to its corresponding analysis plots, including a cumulative distribution of association scores (AUC) (Figure 2B) and a rank-dot plot (Figure 2C). The cumulative distribution of association scores of that TR comparing to all other factors demonstrates the high association scores of many ChIP-seq datasets for that factor. The rank-dot plot shows Irwin-Hall *P*-value scores (−log_10_  *P*-value) against absolute ranks of all TRs with the selected factor highlighted, to demonstrate the overall significance. Users can hover the mouse on other data points to find out which TR it is.

BARTweb also provides download links to all intermediate data files for further exploration, including selected H3K27ac samples from the adaptive lasso regression, the genomic *cis*-regulatory profile and all TR ChIP-seq association scores. A detailed description of each intermediate data file can be found on the Help page.

### BARTweb outperforms existing tools

**Figure 3.**
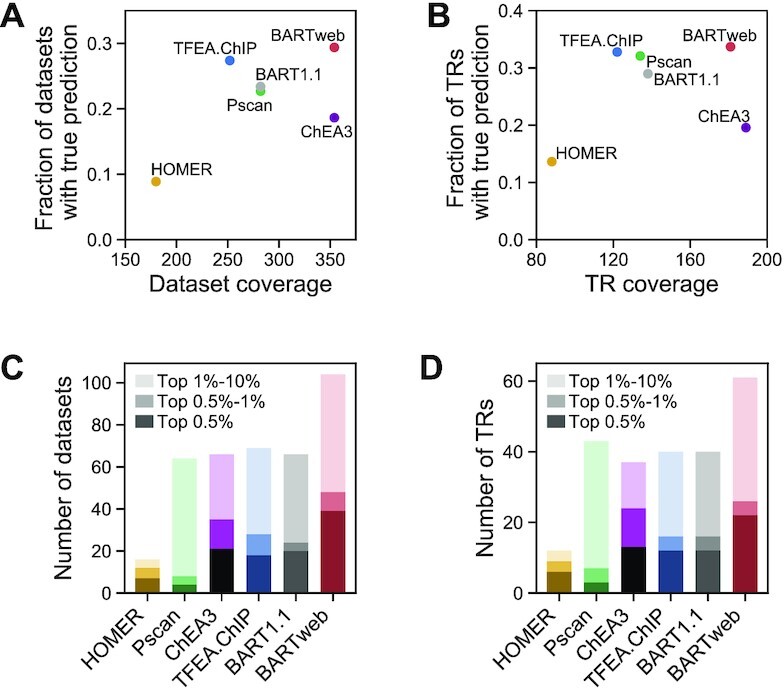
Performance comparison of BARTweb with five other tools on knockTF datasets. (**A**) Performance of each tool reflected by the fraction of knockTF datasets with true prediction (*y*-axis) against the number of knockTF datasets whose KD/KO TR were included in the tool (*x*-axis). (**B**) Performance of each tool reflected by the fraction of unique KD/KO TRs with true prediction (*y*-axis) against the number of unique KD/KO TRs included in the tool (*x*-axis). (**C**) Number of knockTF datasets with true prediction under different rank cutoffs for each tool. (**D**) Number of unique KD/KO TRs with true prediction under different rank cutoffs for each tool.

To evaluate the performance of BARTweb on identifying the correct TRs that regulate an input gene set, we performed TR identification analysis using the gene sets derived from knockTF (28), a database of a comprehensive collection of 570 differential human gene expression profiles with knockdown/knockout (KD/KO) of 308 TFs, and compared the BARTweb results with those generated from several other tools that provide a command line version for batch processing, including BART v1.1 (7), TFEA.ChIP (18), ChEA3 (19), Pscan (16) and HOMER (15) (Supplementary Table S2 and Supplementary Methods). For each differential gene expression profile under KD/KO of a factor, we used a fold-change cutoff of 1.5 to select the up- and down-regulated genes and conduct TR identification analyses separately. If the actual KD/KO factor was ranked among the top 10% of all TRs in the output and the corresponding *P*-value < 0.01 for either up- or down-regulated gene set, we declared that this tool yielded a true prediction on this dataset for this factor. Among the 512 differential expression datasets with at least 100 up- or down-regulated genes (Supplementary Table S3), 354 have their KD/KO factor included in the BARTweb TR library, and BARTweb had true predictions on 104 datasets (29.4%), higher than the other tools (Figure 3A and Supplementary Table S4). If we focused on the number of unique factors that each tool can successfully identify from the konckTF differentially expressed gene sets, BARTweb yielded true predictions for 61 factors (33.7%), also the highest among the tools tested (Figure 3B).

As users usually focus on only a few top-ranked TRs for downstream analysis or validation, we further compared the true prediction performances of the five tools using top 0.5% and top 1% as cutoffs in addition to top 10%. In each case, we found that BARTweb still yielded true predictions on the highest number of gene expression datasets (Figure 3C and Supplementary Table S4) and the highest number of unique factors (Figure 3D). In conclusion, we showed that BARTweb outperforms existing sequence motif-based and ChIP-seq-based tools in identifying regulatory factors using target gene sets from experimental data.

## DISCUSSION

BARTweb is a web server for performing TR association analysis using a large collection of public ChIP-seq data as the sole resource. This approach complements the commonly used sequence motif scan methods for TF identification, and has the unique advantage of utilizing *in vivo* protein–DNA interaction information across the genome for making biologically meaningful discoveries. In addition to sequence-specific TFs, BARTweb can also identify chromatin regulators and some histone variants such as H2A.Z, whose genomic profiles can be measured by ChIP-seq and are included in the data collection.

Utilizing existing ChIP-seq data has become an emerging trend in bioinformatics methodology development for TR analysis. Lisa (29), recently published during preparation of this manuscript, uses a similar integrative modeling approach to build a chromatin model for TR inference. Meanwhile, users should be aware of several limitations of such ChIP-seq data-based TR identification methods, including BARTweb. First, as shown in Figure 3, it is worth noting that most tools can only reach as high as 30% of correct prediction for the knockTF datasets. This might be attributed to both the heterogeneous nature of the data in the knockTF database and the TR coverage of the tools. There is room for further improvement. The prediction power of BARTweb is limited by the range of TRs with existing ChIP-seq data and the data quality. While BARTweb is being maintained and updated, we expect that the TR coverage will grow, as we anticipate that the public ChIP-seq datasets will keep increasing. Second, similar to other tools, BARTweb does not consider cell-type specificity in the TR association analysis. In general, this will not be an issue. Because of the DNA sequence specificity of TF binding, genomic binding profiles of the same TF in different tissue/cell types are usually more similar to each other than binding profiles between different TFs in the same tissue/cell type (7). As a result, as long as the genomic *cis*-regulatory profile correlates with the genomic profile of a regulator, the BART algorithm is still able to find the correct factor, even from a different cell type, but is less likely to identify an irrelevant factor from a relevant cell type.

Last but not least, TR identification process in BARTweb is based on identified peak information from high-quality ChIP-seq data. It is known that a considerable portion of ChIP-seq peaks usually do not contain motif consensus sequences in the regions. This may be because the TR either binds non-canonical motifs or exhibits indirect binding recruited by other factors, or can even be an experimental artifact. These unexplained patterns in ChIP-seq data might create false positive in BARTweb predictions. A more comprehensive characterization of the collected ChIP-seq data, including account for motif-present peaks and motif-absent peaks, might further improve the accuracy of TR analysis. Nevertheless, with a superior performance than several similar tools, BARTweb is an effective and easy-to-use bioinformatics web server for TR analysis for different types of omics data. It can help biologists in gene regulation research interpret various experimental data and develop hypotheses for mechanistic studies.

## DATA AVAILABILITY

BARTweb is freely available at http://bartweb.org; the source code for the updated BART algorithm is available at https://github.com/zanglab/bart2.

## Supplementary Material

lqab022_Supplemental_File

## Notes

Present address: Wenjing Ma, Department of Computer Science, Emory University, Atlanta, GA 30322, USA.

Present address: Ruoyao Shi, Department of Biostatistics, University of Michigan, Ann Arbor, MI 48109, USA.
