# Supplementary material for: BARTweb: a web server for transcriptional regulator association analysis
Source: NAR Genom Bioinform. 2021 Apr 9;3(2):lqab022. doi: 10.1093/nargab/lqab022 (PMC8034776; doi:10.1093/nargab/lqab022)
Supplement: lqab022_Supplemental_File [file lqab022_supplemental_file.pdf]

Supplementary Data

BARTweb: a web server for transcriptional regulator association analysis

Supplementary Data includes 2 Supplementary Figures, 1 Supplementary Algorithm, Supplementary Methods, and 4 Supplementary Tables.

Supplementary Figure S1. BARTweb improvements over BART1.1.

Supplementary Figure S2. BARTweb user input interface.

Supplementary Algorithm 1. Logistic adaptive lasso algorithm.

Supplementary Methods. Scripts and command lines used for running each tool.

Supplementary Table S1. Lists of TRs in human and mouse covered by BARTweb

Supplementary Table S2. Existing tools for TR prediction from input gene set

Supplementary Table S3. List of knockTF datasets used for benchmarking

Supplementary Table S4. Benchmarking results of each tool on TF prediction using knockTF data

Supplementary Figure S1

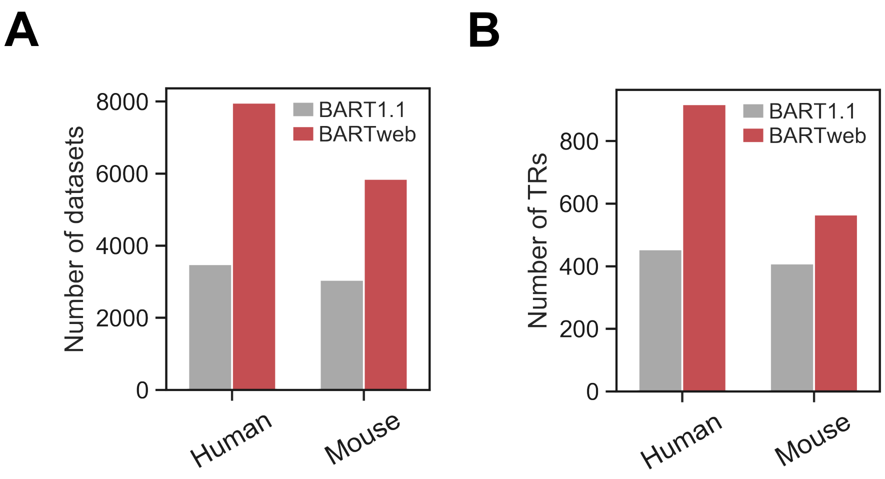

Supplementary Figure S1. BARTweb improvements over BART1.1. (**A,B**) Bar plots showing the number of collected ChIP-seq datasets (**A**) and the number of uniquely covered TRs (**B**) in BARTweb and BART1.1 data libraries.

Supplementary Figure S2

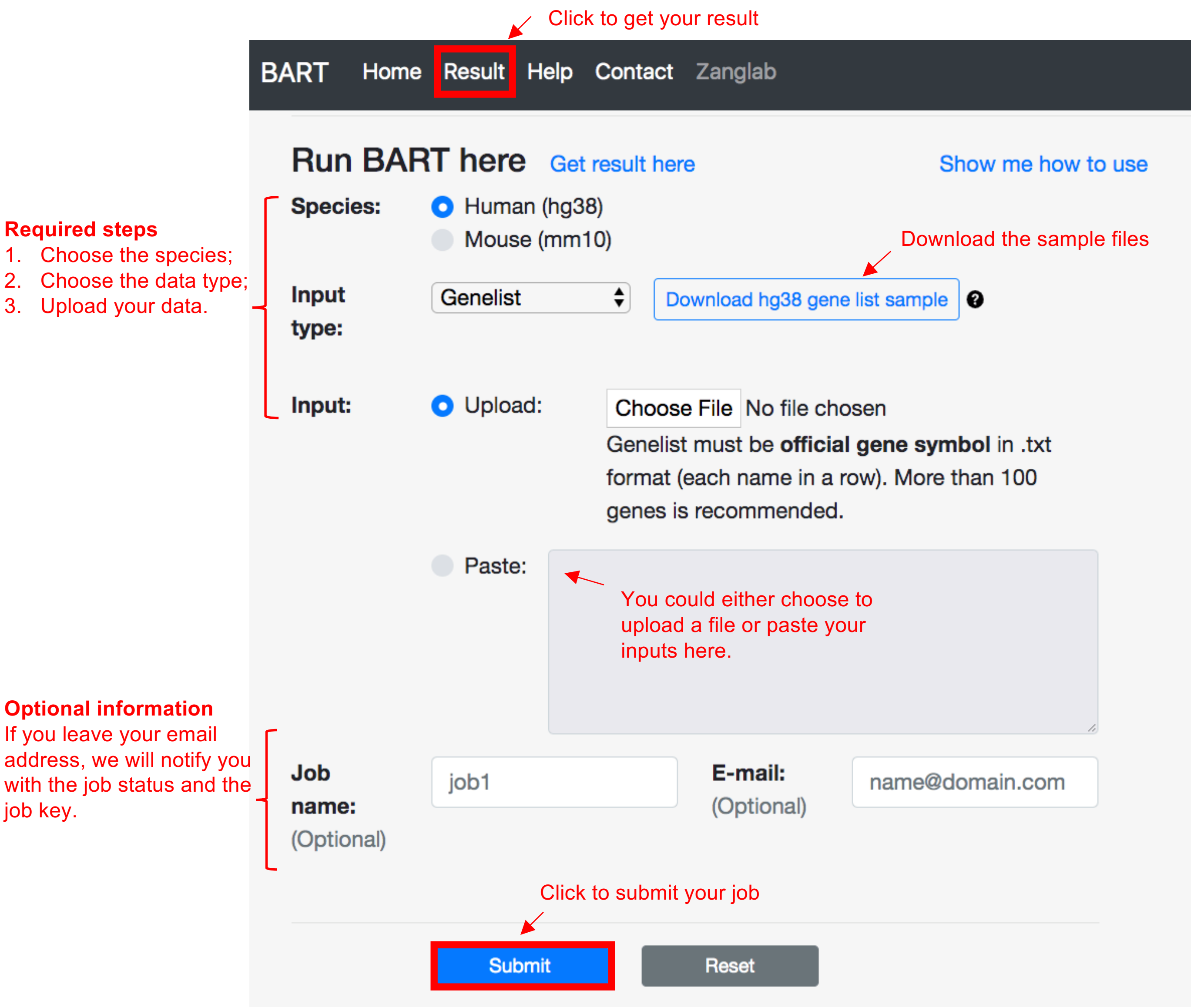

Supplementary Figure S2. BARTweb user input interface. Instructions on how to submit a job are illustrated.

Supplementary Algorithm 1

Algorithm 1: Logistic adaptive lasso

Input: $\boldsymbol{y}, \boldsymbol{P}$
Initialize: $\boldsymbol{w} = \mathbf{1}$
repeat

$\boldsymbol{P}^* = \boldsymbol{P}/\boldsymbol{w}$

$\widehat{\boldsymbol{\beta}} = \arg\min_{\boldsymbol{\beta}} \sum_{i=1}^{n}(-y_i(\boldsymbol{p}_i^{*T}\boldsymbol{\beta}) + \log(1 + e^{\boldsymbol{p}_i^{*T}\boldsymbol{\beta}})) + \lambda \sum_{j=1}^{m}|\beta_j|$

$\widehat{\boldsymbol{\beta}}^* = \widehat{\boldsymbol{\beta}}/\boldsymbol{w}$

$\boldsymbol{w} = 1/2\sqrt{|\widehat{\boldsymbol{\beta}}^*|}$

until *converged*;

Supplementary Methods

Scripts and command lines used for running each tool

BART (version 2.0)

```
/*shell script*/
bart2 geneset -i ${geneset} -s hg38
```

HOMER (version 4.11)

```
/*shell script*/
findMotifs.pl ${geneset} human ${output} -p 4
```

Pscan (version 1.5)

```
/*shell script*/
fai_file=Homo _sapiens_1000up_0down.fasta.fai
fasta_file=Homo_sapiens_1000up_0down.fasta
matrix_file=Homo_sapiens_1000_0.short_matrix
cat ${fai_file} |cut -f1 |sort -u > ${fai_file}.uniq
convertIDs.pl ${geneset} human refseq > ${geneset}.refseq
cat ${geneset}.refseq |awk '{print "hg38_refGene_"$0}' > ${geneset}.refseq.txt
cat ${fai_file}.uniq ${geneset}.refseq.txt |sort|uniq -d > ${geneset}.refseq.txt.fai
xargs samtools faidx ${fasta_file} < ${geneset}.refseq.txt.fai > ${geneset}.fa
pscan -q ${geneset}.fa -m ${matrix_file}
```

ChEA3 (version 3)

```
/*R script*/
library(httr)
library(jsonlite)
genes <-read.table(${geneset}),stringsAsFactors=FALSE)
url = "https://amp.pharm.mssm.edu/chea3/api/enrich/"
encode = "json"
payload = list(query_name = "myQuery", gene_set = genes[,1])
response = POST(url = url, body = payload, encode = encode)
json = content(response, "text")
results = fromJSON(json)
write_json(results,file.path(outdir,paste(allfiles[i],".json",sep="")))
results_csv <- data.frame(TF = results$"Integrated--meanRank"$"TF", Score =
results$"Integrated--meanRank"$"Score")
write.csv(results_csv,file = ${output},row.names=TRUE)
```

TFEA.ChIP (version 1.10)

```
/*R script*/
library(TFEA.ChIP)
genes <-read.table((${geneset}),stringsAsFactors=FALSE)
cont_genes <-contingency_matrix(genes[,1])
results <- getCMstats(cont_genes)
write.csv(results,file = ${output},row.names=TRUE)
```
